# Disulfiram, an Anti-alcoholic Drug, Targets Macrophages and Attenuates Acute Rejection in Rat Lung Allografts

**DOI:** 10.3389/ti.2024.12556

**Published:** 2024-04-08

**Authors:** Nobuyuki Yoshiyasu, Rei Matsuki, Masaaki Sato, Hirokazu Urushiyama, Etsuko Toda, Yasuhiro Terasaki, Masaki Suzuki, Aya Shinozaki-Ushiku, Yuya Terashima, Jun Nakajima

**Affiliations:** ^1^ Department of Thoracic Surgery, Graduate School of Medicine, The University of Tokyo, Tokyo, Japan; ^2^ Department of Respiratory Medicine, Graduate School of Medicine, The University of Tokyo, Tokyo, Japan; ^3^ Department of Thoracic Surgery, The University of Tokyo Hospital, Tokyo, Japan; ^4^ Department of Respiratory Medicine, The University of Tokyo Hospital, Tokyo, Japan; ^5^ Department of Analytic Human Pathology, Nippon Medical School, Tokyo, Japan; ^6^ Division of Molecular Regulation of Inflammatory and Immune Diseases, Research Institute for Biomedical Sciences (RIBS), Tokyo University of Science, Chiba, Japan; ^7^ Division of Pathology, Nippon Medical School Hospital, Tokyo, Japan; ^8^ Department of Pathology, The University of Tokyo Hospital, Tokyo, Japan

**Keywords:** lung transplantation, acute lung rejection, macrophage, disulfiram, rodent study

## Abstract

Macrophages contribute to post-transplant lung rejection. Disulfiram (DSF), an anti-alcoholic drug, has an anti-inflammatory effect and regulates macrophage chemotactic activity. Here, we investigated DSF efficacy in suppressing acute rejection post-lung transplantation. Male Lewis rats (280–300 g) received orthotopic left lung transplants from Fisher 344 rats (minor histocompatibility antigen-mismatched transplantation). DSF (0.75 mg/h) monotherapy or co-solvent only (50% hydroxypropyl-β-cyclodextrin) as control was subcutaneously administered for 7 days (n = 10/group). No post-transplant immunosuppressant was administered. Grades of acute rejection, infiltration of immune cells positive for CD68, CD3, or CD79a, and gene expression of monocyte chemoattractant protein and pro-inflammatory cytokines in the grafts were assessed 7 days post-transplantation. The DSF-treated group had significantly milder lymphocytic bronchiolitis than the control group. The infiltration levels of CD68^+^ or CD3^+^ cells to the peribronchial area were significantly lower in the DSF than in the control groups. The normalized expression of chemokine ligand 2 and interleukin-6 mRNA in allografts was lower in the DSF than in the control groups. Validation assay revealed interleukin-6 expression to be significantly lower in the DSF than in the control groups. DSF can alleviate acute rejection post-lung transplantation by reducing macrophage accumulation around peripheral bronchi and suppressing pro-inflammatory cytokine expression.

## Graphical Abstract

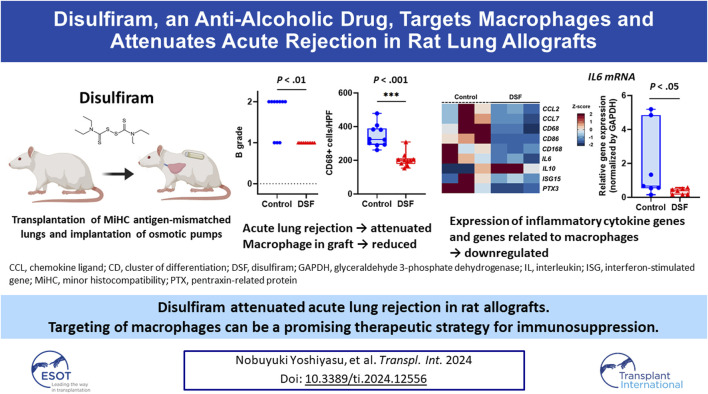

## Introduction

Lung transplantation is an established therapy for end-stage lung disease. However, lung rejection remains the most challenging complication after transplantation. Acute lung rejection can be a risk factor for developing chronic lung allograft dysfunction (CLAD) even after a single episode [1, 2]. Thus, preventing acute rejection may improve long-term survival by preventing the development of CLAD. Despite using common maintenance immunosuppressive drugs, such as calcineurin inhibitors, anti-metabolites, and steroids, over 25% of lung transplant recipients experience acute rejection at least once within a year after transplantation [1, 3]. In recent years, a few research groups have proposed that not only T cells, but also macrophages are involved in the development of acute lung rejection [4–6]. Cell profiles during acute rejection obtained using single-cell RNA sequencing (RNA-Seq) of human samples provide evidence of macrophage involvement [7].

Disulfiram (DSF), a well-known anti-alcoholic drug [8], also shows other pharmacological effects, such as anti-inflammatory and anti-cancer effects [9–12]. Furthermore, DSF inhibits the activity of the cytoplasmic protein FROUNT, which regulates the chemotactic signals of macrophages [12, 13]. Owing to the broad therapeutic potential of DSF, repositioning of DSF has garnered interest recently. Drug repositioning is the process of discovering new indications for approved or failed drugs [14]. Clinical trials on the use of DSF for various diseases, such as coronavirus disease 2019, human immunodeficiency virus infection, and treatment-refractory multiple myeloma have been conducted or are ongoing [15]. However, there are no reports on the therapeutic efficacy of DSF in acute post-transplant rejection. We hypothesized that DSF could attenuate acute lung rejection by suppressing the chemotaxis of macrophages to allografts after lung transplantation. Therefore, in this study, we aimed to investigate the efficacy of DSF in a rat model of acute rejection after orthotopic lung transplantation.

## Materials and Methods

### Animal Models

This study was approved by the Experimental Animal Ethics Committee of the University of Tokyo under license number H20-204 (issued January 19, 2021). All procedures complied with the Institutional Animal Care and Use Committee Guidelines of the University of Tokyo. Specific-pathogen-free inbred male rats were purchased from Japan SLC, Inc. (Hamamatsu, Japan). All rats (age: 12–13 weeks; weight: 280–300 g) received adequate care according to the animal study protocols. The animal experiments were conducted using Lewis (LEW; RT1^l^) and Fischer 344 (F344; RT1^lv1^) rats in accordance with the guidelines. Allogenic orthotopic left lung transplantation was performed using the modified cuff technique as reported previously [16]. F344 rats were used as donors, whereas LEW rats were used as recipients in the minor histocompatibility (MiHC) antigen-mismatched transplantation procedure.

### Preparation of DSF Solution

DSF (Mitsubishi Tanabe Pharma, Osaka, Japan) was dissolved in 50% hydroxypropyl-β-cyclodextrin (HBC) (Tokyo Chemical Industry, Tokyo, Japan) with agitation to a final concentration of 37.5 mg/mL, and it was stored at 4 °C under a light shield. ALZET osmotic pumps (model 2ML1; DURECT, Cupertino, CA, United States), which deliver solutions continuously at a rate of 10 μL/h for 7 days, were filled with 2 mL of the DSF solution or 50% HBC per piece just before implantation. The pumps were unlabeled; hence, the operator was blinded to the content of each pump.

### Treatment Protocols

**FIGURE 1:**
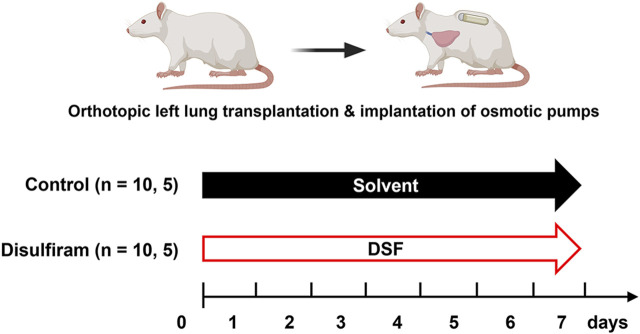
Treatment intervention protocols with osmotic pumps for 7 days. Minor histocompatibility complex-mismatched left lung transplantation was performed from Fisher 344 rats to Lewis rats. The control group was administered 50% hydroxypropyl-β-cyclodextrin as a solvent and disulfiram was administered to the treatment group. Samples such as lung tissues (n = 10/group) and bronchoalveolar lavage fluid (n = 5/group) were collected on post-operative day 7. DSF, disulfiram.

The treatment protocols for the recipients are summarized in Figure 1. Prior to making the skin incision, methylprednisolone sodium (10 mg per animal unit; SHIONOGI, Osaka, Japan) and cefazoline sodium (10 mg per animal unit; Nipro Medical, Osaka, Japan) were injected subcutaneously or peritoneally into the recipients to prevent reperfusion injury and infection, respectively. These injections were administered under general anesthesia. It is important to note that the recipients did not receive any post-transplant immunosuppressive drugs. After reperfusion, two osmotic pumps, primed with 50% HBC (control group, n = 10) or DSF solution (DSF group, n = 10), were subsequently embedded under the skin of each recipient. The recipients were euthanized on day 7 post-transplantation. The DSF group rats were administered 18 mg DSF/day until sacrifice, equivalent to approximately 600 mg/day in humans. All rats had *ad libitum* access to water throughout the study. Recipient feeding was fixed at 200 g for 7 days and body weight was measured daily.

### Histopathological Evaluation and Immunohistochemical Staining

**FIGURE 2:**
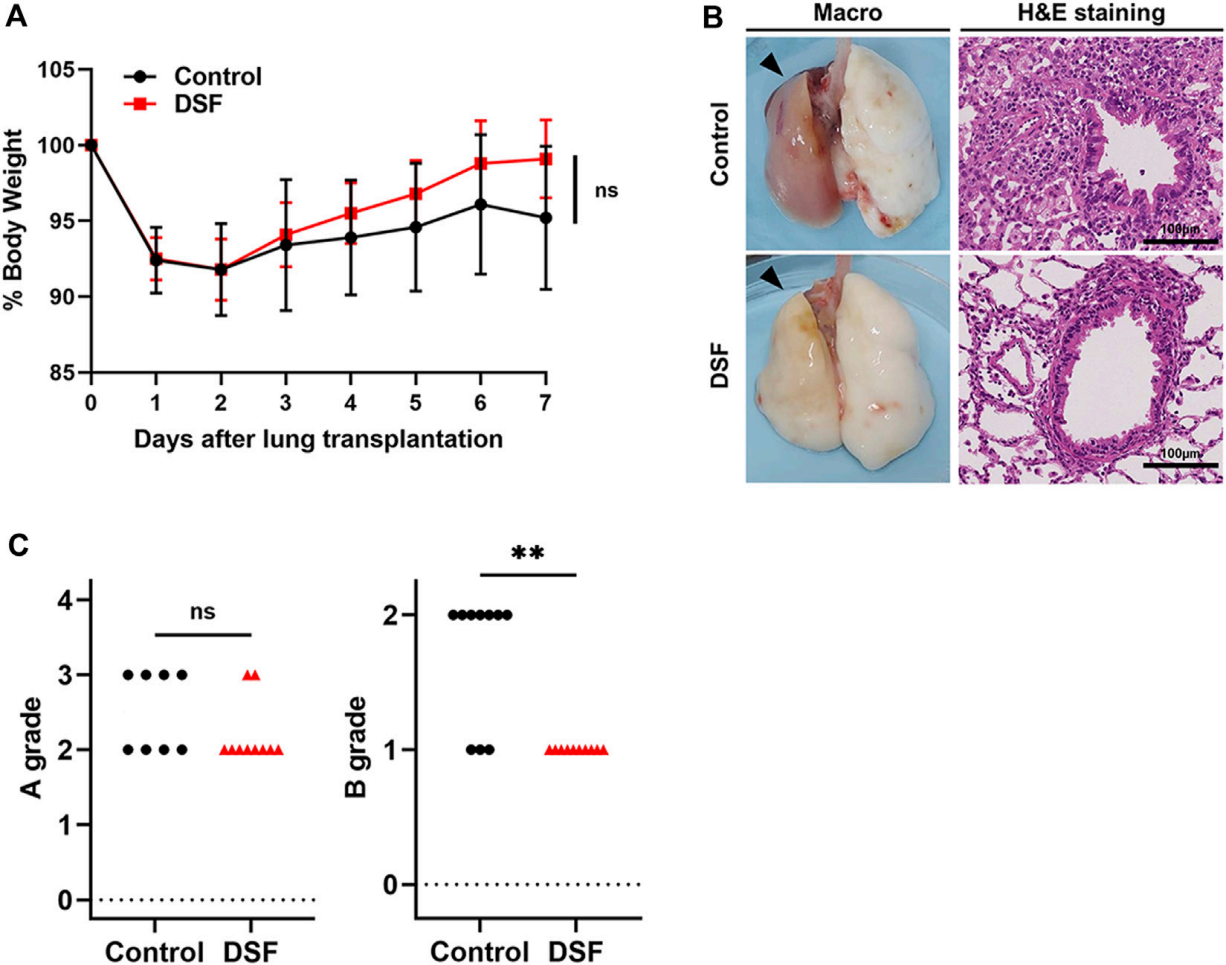
Physical and histopathological effects of disulfiram. **(A)** % Body weight. Daily body weight was measured, and it is indicated as a percent of that right after surgery. **(B)** Macroscopic (black arrows denote allografts) and microscopic images (H&E staining; high-power field, magnification ×400). Scale bar: 100 µm. **(C)** A/B grading of acute lung rejection. The scores were settled according to the criteria of the International Society for Heart and Lung Transplantation. Two cases in the control group were excluded because of AX. ns, not significant. **p < 0.01. DSF, disulfiram; H&E, hematoxylin and eosin.

**FIGURE 3:**
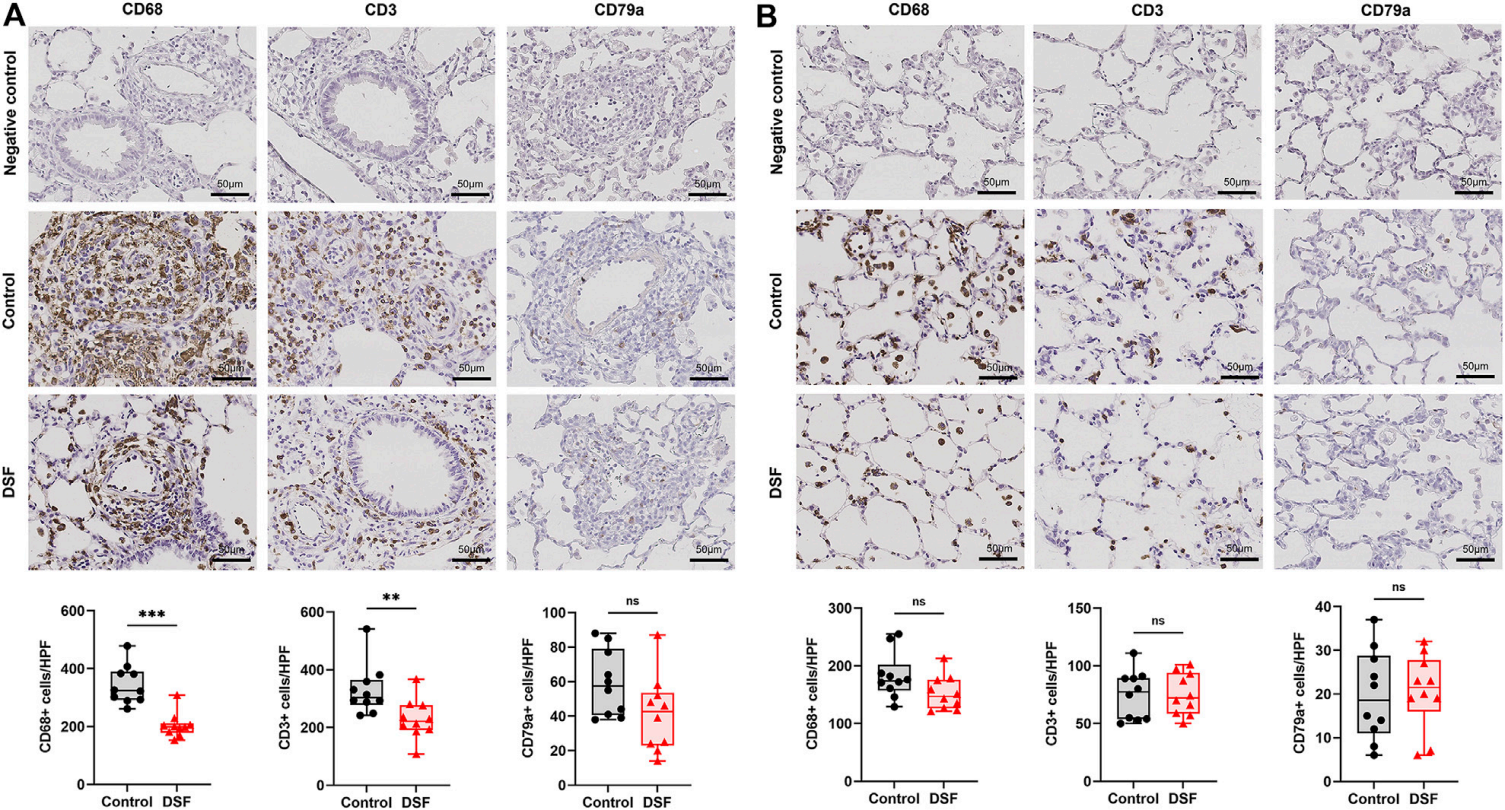
Immunohistochemical staining. The plots show the counts of CD68-, CD3-, and CD79a-positive cells in the negative control, control, and disulfiram (DSF) groups per high-power field (HPF; magnification, ×400). **(A)** Perivascular/peribronchiolar area. **(B)** Alveolar area. The box-and-whiskers dot plots represent the medians and interquartile ranges with the minimum and maximum values. ns, not significant. **p < 0.01; ***p < 0.001.

The cranial sections (approximately two-thirds) of the allograft were fixed in 10% formalin (FUJIFILM Wako Pure Chemical, Osaka, Japan) and embedded in paraffin. The sections were stained with hematoxylin–eosin. According to the criteria of the International Society for Heart and Lung Transplantation for acute lung rejection, expert pathologists (M.S. and A.U.) graded sections A (subtypes: 0–4, X) when they observed infiltration of perivascular mononuclear cells or B (subtypes: 0–2R, X) when they observed lymphocytic bronchiolitis, in a double-blinded fashion (Figure 2) [17]. The extent of perivascular inflammation, referred to as A-grade, is determined by examining the infiltration of mononuclear cells around vascular structures, within the interstitial spaces of the submucosa, and along the alveolar partitions. This is systematically categorized into various levels: A0 (none), A1 (minimal), A2 (mild), A3 (moderate), A4 (severe), and AX (ungradable). Additionally, the evaluation of airway inflammation, designated as B-grade rejection, focuses on the lymphocytic activity within the bronchiole submucosa. The extent of this response is classified into the following distinct categories: B0 (none), B1R (low grade), B2R (high grade), and BX (ungradable). Particularly, when lymphocyte infiltration beyond the basement membrane was observed, the more advanced stage B2R was graded. For immunohistochemistry (IHC), sections were deparaffinized and incubated with 0.1% pepsin for 40 min at 37 °C for CD3 and CD68 staining, and with 0.01 M citrate buffer at a pH of 6.0 for 20 min at 120 °C for CD68 staining. This was followed by overnight incubation with the following primary antibodies: anti-CD3 (rabbit polyclonal; 1:300; DAKO, Tokyo, Japan), anti-CD79a (mouse monoclonal; 1:100; Biocare Medical, Pacheco, CA, United States), and anti-CD68 (mouse monoclonal; 1:1000; BMA Biomedicals, Augst, Switzerland). Histofine Simple Stain Rat MAX PO (MULTI; Nichirei Bioscience, Tokyo, Japan) was used as the secondary antibody, and 3,3′-diaminobenzidine (DOJINDO, Kumamoto, Japan) was used for detection. The primary antibodies were omitted to serve as negative controls for each CD staining, and assessments were conducted to detect false positives. The sections were counterstained with hematoxylin. In the IHC evaluation, six high-power field images (magnification ×400) were randomly chosen from each section and the positive cell counts per field were automatically determined using a hybrid cell count application (BZ-H4C; KEYENCE, Osaka, Japan) in BZ-X Analyzer software (BZ-H4A; KEYENCE). We separately conducted our analyses of the perivascular/peribronchiolar area when grading the extent of rejection or the alveolar area without vascular and bronchial structures (Figure 3).

### Transcriptome Analysis via RNA-Seq

**FIGURE 4:**
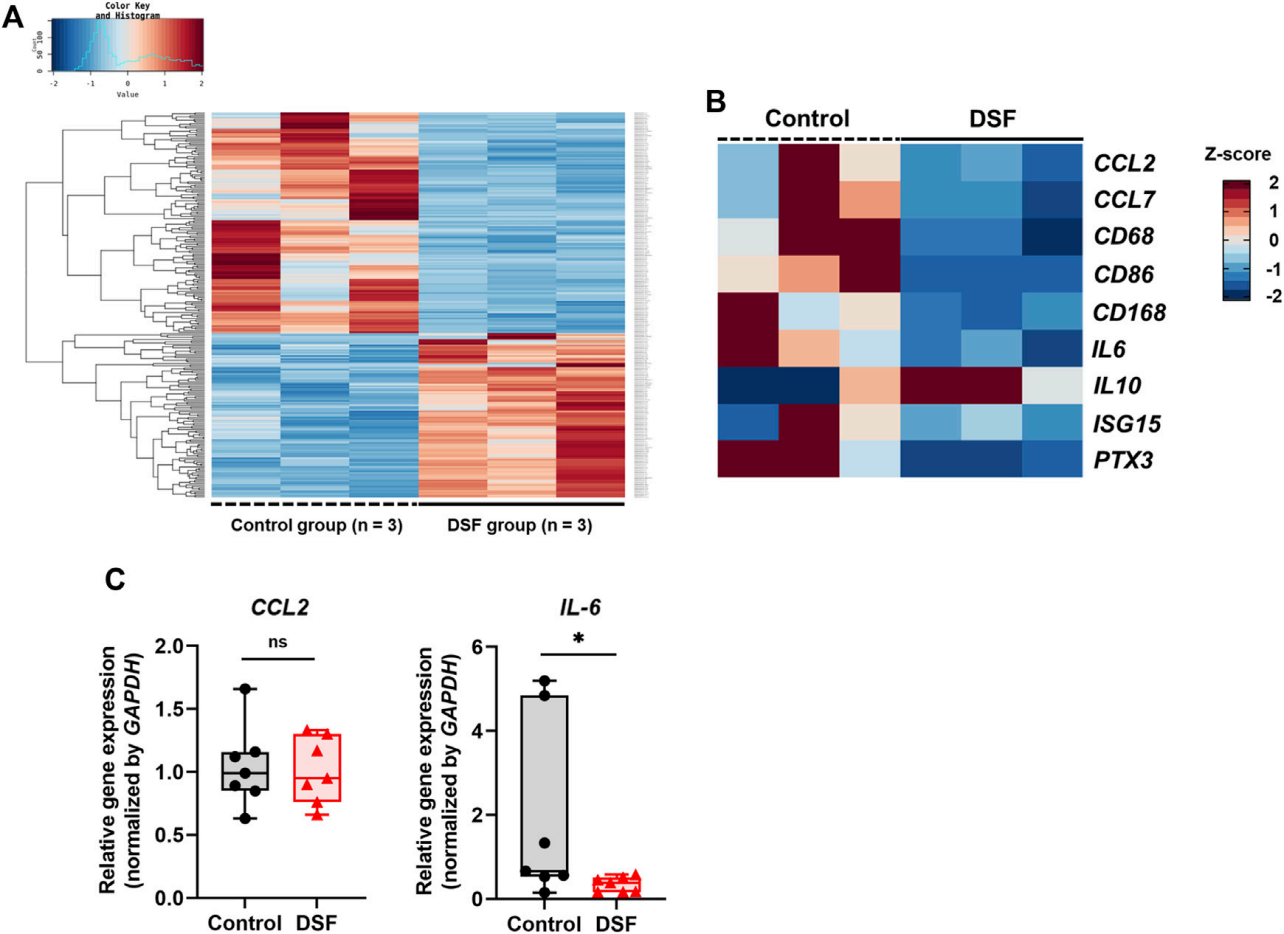
Gene expression analyses using RNA sequencing (RNA-Seq) and real-time quantitative polymerase chain reaction (RT-qPCR). **(A)** Heat map based on the z-scores of differentially expressed genes between the control and disulfiram (DSF) groups (n = 3/group). **(B)** Extracted heat map focusing on the genes related to macrophages and acute rejection. **(C)** RT-qPCR analysis to validate the RNA-Seq results. The expression of *CCL2* and *IL-6* (n = 7 each) was normalized with that of *GAPDH*. The box-and-whiskers dot plots represent the medians and interquartile ranges with the minimum and maximum values. ns, not significant. *p < 0.05.

We selected representative rejection cases based on histopathological findings (n = 3/group) for RNA-Seq. Total RNA was extracted from the frozen samples, that is, the caudal one-third of the allografts, using ISOSPIN Cell and Tissue RNA (Nippon Gene, Tokyo, Japan). RNA quality was checked using Agilent 4150 TapeStation (Agilent Technologies, Santa Clara, CA, United States). A strand-specific RNA library was prepared using 1 µg of each sample with the NEBNext Poly(A) mRNA Magnetic Isolation Module (NEB, Ipswich, MA, United States) and NEBNext Ultra II Directional RNA Library Prep Kit (NEB). RNA sequences were obtained using paired-end reads (150 bp × 2) on the NovaSeq 6000 platform (Illumina, San Diego, CA, United States). Differentially expressed genes (DEGs) between the control and DSF groups were identified with the cut-off criteria |log_2_ fold change| > 1 and *Q*-value <0.05 using DESeq2 software^1^. Raw read counts were normalized using the relative log expression method (Figure 4). Heat maps with z-scores of the normalized gene expression were created using all genes that matched the criteria^2^. Ward’s clustering method and correlation distances were also used to generate hierarchical clusters of genes from the generated heat maps. To further examine the potential biological roles of the DEGs affected by DSF, we conducted a Gene Ontology (GO) term enrichment analysis using the DAVID WebService package^3^.

### Validation Using Real-Time Quantitative Polymerase Chain Reaction

The remaining samples (n = 7/group) were used to validate the transcription levels obtained via RNA-seq using real-time quantitative polymerase chain reaction (RT-qPCR). Relative expression of *CCL2* and *IL-6* in both groups was normalized against the expression level of the internal control gene glyceraldehyde 3-phosphate dehydrogenase (*GAPDH*). Total RNA (1 μg) isolated from samples was used for reverse transcription with the High-Capacity RNA-to-cDNA™ Kit (Thermo Fisher Scientific, Waltham, MA, United States) in a 20 μL volume. The protocol involved incubating at 37 °C for 60 min, followed by heating to 95 °C for 5 min, and finally cooling to 4 °C. RT-qPCR was then performed on the Applied Biosystems^®^ 7500 System (Thermo Fisher Scientific) using 100 ng of cDNA and the TaqMan™ Gene Expression Master Mix (Thermo Fisher Scientific). Each sample was processed in duplicate. The PCR conditions were as follows: an initial 2-min step at 50 °C, a 10-min step at 95 °C, followed by 40 cycles of 15 s at 95 °C and 1 min at 60 °C, concluding with a cooldown to 25 °C. Data analysis was conducted using the 7500 System SDS Software Version 1.4 (Thermo Fisher Scientific). The following probes were used for RT-qPCR: *CCL2* (NM_031530), *GAPDH* (NM_017008), and *IL-6* (NM_012589). For the negative controls, a no-template control from the RT reaction and a no-template control from the RT-qPCR reaction were used. Relative gene expression was calculated using the comparative ΔΔCT method [18].

### Bronchoalveolar Lavage Fluid (BALF) Collection

Additional rats (n = 5/group) were subjected to left lung transplantation and implantation of osmotic pumps to obtain BALF samples. Briefly, their tracheas were cannulated, and lungs were lavaged thrice with 3 mL of phosphate-buffered saline on day 7 post-transplantation. The LUNA-FL Dual Fluorescence Cell Counter (Logos Biosystems, Gyeonggi-do, South Korea) was used to measure total cell count (TCC). Smears stained with Diff Quick were then used by pulmonologists to assess cell fractions in a double-blinded manner.

### Determination of Pro-inflammatory Cytokine Levels and Potent Chemokines for Macrophages

On post-operative day (POD) 7, blood samples (3 mL) were collected from the inferior vena cava of the rats before heparinization and centrifuged to obtain sera (2,500 *g*, 10 min, 21 °C). The sera (n = 10 each), and the remaining BALF (n = 5 each; additional rats) after centrifugation (3,200 *g*, 20 min, 4 °C) were preserved at −80 °C. A MILLIPLEX MAP Kit Rat Cytokine/Chemokine Magnetic Bead Panel (MilliporeSigma, Burlington, VT, United States) was used to measure the protein concentrations in the serum and BALF. The levels of the following cytokines and chemokines were measured: chemokine ligand (CCL)2, interleukin (IL)-1β, IL-6, interferon-γ, and tumor necrosis factor-α.

### Statistical Analysis

Continuous variables are presented as medians and interquartile ranges, except for % body weight, which is presented as the mean ± standard deviation due to its normal distribution. Mann–Whitney *U* test or Student’s t-test was used to compare the values, respectively. Analyses were performed using R software (version 4.2.1; R Foundation for Statistical Computing, Vienna, Austria). The Benjamini–Hochberg method was used to identify DEGs. GraphPad Prism (version 9; GraphPad Software, San Diego, CA, United States) was used for creating figures. *p* < 0.05 or *Q* < 0.05 indicated significant differences in two-tailed tests.

## Results

### Weight Changes

The percentage of rats’ weights after treatment to the baseline value (% body weight) is shown in Figure 2. Both groups showed weight loss for 2 days with gradual recovery thereafter. Specifically, the % body weight was 95.2% ± 4.7% in the control group and 99.1% ± 3.6% in the DSF group on POD 7 (*p* = 0.052; Figure 2A).

### Histological Findings

On POD 7, allogenic transplanted lungs in rats treated with DSF had a more whitish appearance and milder rejection than the control (Figure 2B). Perivascular lymphocytic infiltration (*p* = 0.321; Figure 2C) was not significantly altered, while lymphocytic bronchiolitis (*p* = 0.0031; Figure 2C) was significantly milder in the DSF group than in the control group. In the perivascular/peribronchiolar area, the infiltration of CD68^+^ and CD3^+^ cells was significantly inhibited after DSF treatment (*p* = 0.0001 and *p* = 0.0029, respectively; Figure 3A). In the alveolar area, the proportions of infiltrating CD68^+^, CD3^+^, and CD79a+ cells were not reduced after DSF treatment (Figure 3B). No false positives were observed in any of the CD staining instances.

### Differential Gene Expression Analysis

In the DEG analysis between the control and DSF groups, 258 genes that matched the cut-off criteria (|log_2_ fold change | > 1.0, and *Q* < 0.05) were identified from RNA-Seq analysis. The expression heat map of DEGs indicated that 134 genes were downregulated after DSF treatment (Figure 4A). Among them, the expression of genes associated with macrophages and acute lung rejection was downregulated in the DSF group compared with that in the control group (Figure 4B). The expression of *CD86* and *CD163* was significantly downregulated in the DSF group compared with that in the control group (*Q* = 0.002 and *Q* = 0.014, respectively; Supplementary Figure S1). The expression of *IL-6* was significantly downregulated in the DSF group compared with that in the control group (*Q* = 0.037; Supplementary Figure S1), and these findings are consistent with the RT-qPCR results (*p* = 0.047; Figure 4C). Additionally, the expression of the monocyte chemotactic protein CCL2 was lower in the DSF group than in the control group (*Q* = 0.100; Supplementary Figure S1); however, the RT-qPCR analysis showed only a slight change in its expression in both groups (*p* = 0.874; Figure 4C). When the cut-off value was increased to 1.5-fold higher expression (|log_2_ fold change| > 0.6), upregulation of IL-10 expression was observed in the DSF group, but the difference between the groups was not significant (*Q* = 0.654; Supplementary Figure S1). No-template controls exhibited undetermined Ct values, indicating the absence of detectable amplification.

### GO Analysis

**FIGURE 5:**
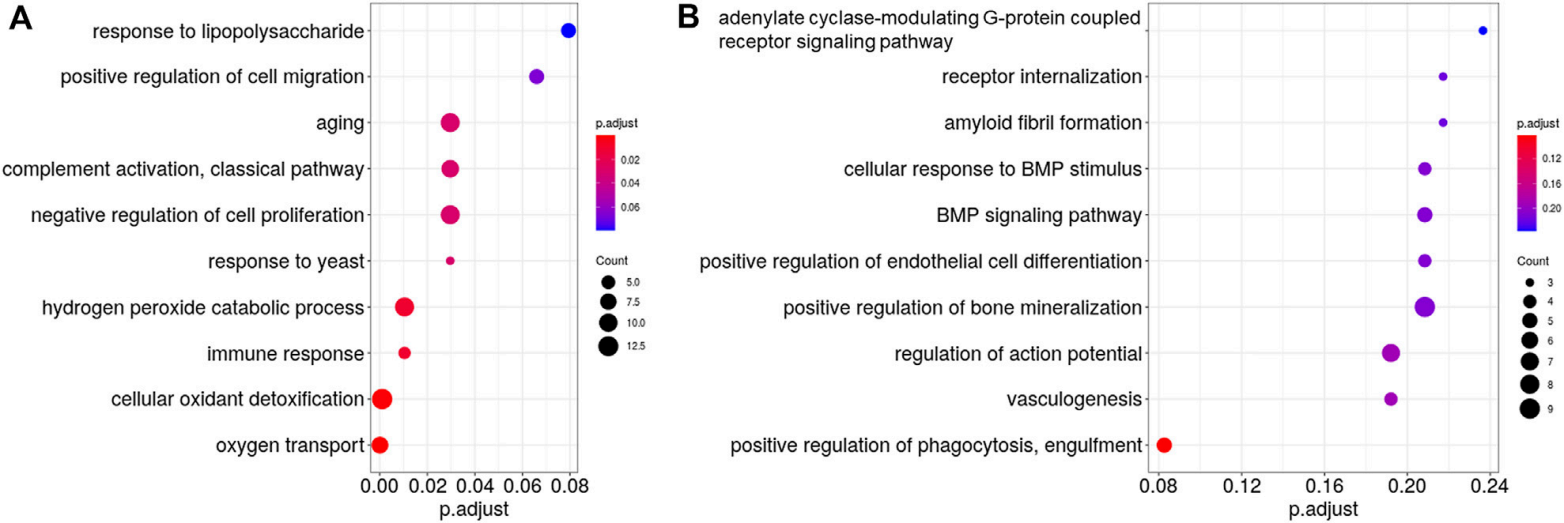
Gene Ontology (GO) analysis of the differentially expressed genes between the control and disulfiram (DSF) groups. The top 10 most enriched GO terms (biological process) of **(A)**, downregulated genes and **(B)**, upregulated genes after DSF treatment. The vertical axis shows the GO terms, whereas the horizontal axis shows the adjusted *p*-values (*Q*-values). Gradations are applied according to the adjusted *p*-values. Circles represent the gene counts related to each GO term. If the GO terms had the same adjusted *p*-value, they are listed alphabetically from top to bottom.

The downregulated genes in the DSF group were significantly enriched in eight biological process terms (Figure 5A), the top five being oxygen transport (GO:015671; *Q* < 0.001), cellular oxidant detoxification (GO:0098869; *Q* < 0.001), hydrogen peroxide catabolic process (GO:0042744; *Q* = 0.0104), immune response (GO:0006955; *Q* = 0.0104), and aging (GO:0007568; *Q* = 0.0296). In contrast, the upregulated genes were not significantly enriched in any biological process (Figure 5B).

### TCC and Cell Fractionation in the BALF

**FIGURE 6:**
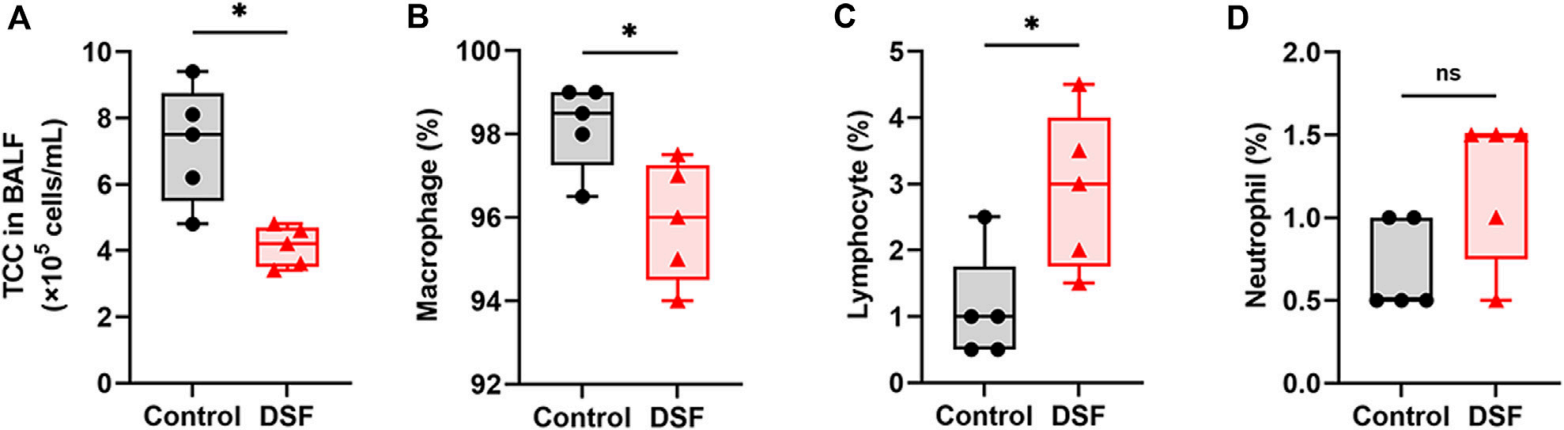
Bronchoalveolar lavage fluid and their cell profiles. **(A)** Total cell count per milliliter of bronchoalveolar lavage fluid. **(B)** Macrophages (%), **(C)**, lymphocytes (%), and **(D)**, neutrophils (%). The box-and-whiskers dot plots represent the medians and interquartile ranges with the minimum and maximum values. ns, not significant. *p < 0.05. BALF, bronchoalveolar lavage fluid; DSF, disulfiram; TCC, total cell count.

The TCC in the BALF was markedly lower in the DSF group than in the control group (*p* = 0.0159; Figure 6A). The cell profile of the DSF group showed that the proportion of macrophages significantly decreased (*p* = 0.032; Figure 6B), whereas the percentage of lymphocytes significantly increased (*p* = 0.024; Figure 6C) compared with that in the control group. There was no difference in the proportion of neutrophils between the groups (*p* = 0.143; Figure 6D). In terms of the absolute counts in the BALF (Supplementary Figure S2), macrophages in the DSF group significantly decreased compared to those in the control group (median: 3.8 × 10^5^ vs. 7.3 × 10^5^ cells/mL; *p* = 0.008). Conversely, there was no significant difference in lymphocyte counts between the DSF and control groups (median: 9.9 × 10^3^ vs. 4.0 × 10^3^ cells/mL; *p* = 0.151).

### Protein Concentrations in the Serum and BALF

**FIGURE 7:**
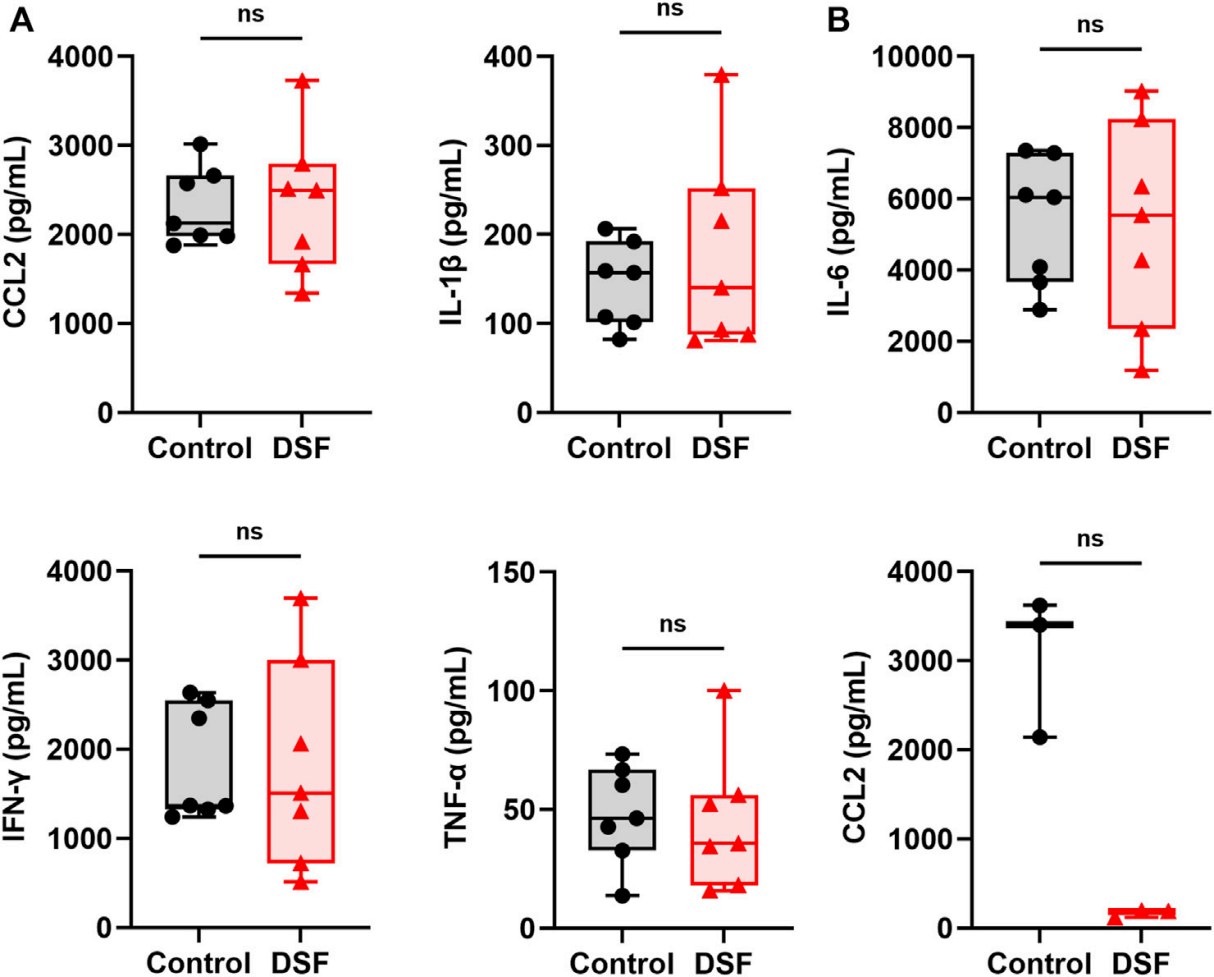
Cytokines and chemokines in **(A)**, serum (n = 7 each) and **(B)**, bronchoalveolar lavage fluid (n = 3 each). The box-and-whiskers dot plots represent the medians and interquartile ranges with the minimum and maximum values. ns, not significant. CCL2, chemokine ligand 2; DSF, disulfiram; IFN-γ, interferon-gamma; IL-1β, interleukin-1 beta; IL-6, interleukin-6; TNF-α, tumor necrosis factor α.

The median concentration of CCL2 in the serum was 2,123 pg/mL in the control group and 2,493 pg/mL in the DSF group, and the difference between the groups was not significantly different (*p* = 0.805; Figure 7A). Among the measurable samples (n = 3 each), the CCL2 level in the BALF was relatively lower in the DSF group than in the control group (median: 3,400 pg/mL vs. 189 pg/mL; *p* = 0.100; Figure 7B). The levels of other cytokines in the serum did not significantly change after DSF treatment (Figure 7A), and they were undetectable in the BALF of both groups.

## Discussion

In this study, we demonstrated that DSF could attenuate acute rejection after MiHC lung transplantation in rats without using immunosuppressants. DSF reduced the accumulation of macrophages and T cells around the bronchioles in allografts, which might contribute to the prevention of bronchiolitis obliterans (BO). Furthermore, the expression of genes associated with macrophages and inflammatory cytokines in the lungs was downregulated after DSF treatment. These results support our hypothesis that macrophages are involved in acute rejection after lung transplantation and that DSF suppresses their chemotaxis.

The direct allorecognition of T cells is generally observed in acute lung rejection cases [19, 20]. DSF may have the potential to inhibit allorecognition and suppress macrophage migration and activation. Following lung transplantation, cells of the acquired immune system in recipients are mobilized to the graft by recognizing alloantigens presented by the donor’s antigen-presenting cells [21]. In addition, monocyte-derived macrophages could migrate and cause injury to the graft together with T cells because they depend on the microenvironment and are particularly plastic [6, 22]. The present study showed that the post-operative regimen of DSF monotherapy for 7 days resulted in a reduction of lymphocytic bronchiolitis and decrease in the number of CD68^+^ and CD3^+^ cells in the perivascular/parabronchial area. The reduced accumulation of immunocompetent cells was presumably associated with the DSF-induced inhibition of their mobilization from circulation. Similarly, some animal studies have also shown that inhibiting macrophage migration to the allograft suppressed acute lung rejection [4, 5, 23]. Furthermore, single-cell RNA-Seq data of the BALF from humans with acute rejection of the lungs and biopsy samples of lungs with chronic rejection suggested the involvement of macrophages [7]. These findings strongly indicate the involvement of macrophages in lung transplant rejection.

Our group reported that DSF inhibits the expression of the cytoplasmic protein FROUNT in macrophages, suppressing their migration and activation [12, 13], whose effect may have decreased the proportion of CD68^+^ cells in the grafts. We did not observe a decrease in CCL2 levels in the serum or lung tissues in this study, and this is consistent with the fact that DSF has been shown to inhibit intracellular signaling between FROUNT and chemokine receptors (CCR2 or CCR5) on macrophages [24]. A previous study showed that when CCR2-positive cells accumulated in the inflamed lung, CCL2 was consumed in the serum and lung tissue [25]. Conversely, another study reported that CCL2 levels in the serum and grafts were higher in CCR2-deficient recipients than in wild-type recipients [26]. The results of these studies support a part of our results.

Repositioning for DSF has been proposed since it also has other therapeutic benefits, such as anti-inflammatory and anti-cancer effects [9–12]. However, there have been no reports on the preventive effect of DSF on rejection after solid organ transplantation. In a previous study, RNA-Seq of samples of rodents infected with severe acute respiratory syndrome coronavirus 2 and then administered DSF revealed the downregulation of the immunity pathway and complement and coagulation cascade [27]. The GO analysis in the present study also showed similar findings. Therefore, DSF is considered to have the potential to downregulate the immune response, attenuating organ and tissue rejection.

The number of macrophages in the alveolar area was not significantly different after DSF treatment compared with that in the perivascular/peribronchial area. This may have the advantage of maintaining their activity against bacteria and viruses to alveolar invasion. The imbalance in drug efficacy between areas can result from the main rejection site being a perivascular/peribronchial area and the differences in the turnover rates of tissue-resident and monocyte-derived macrophages. Monocyte-derived macrophages are produced from the bone marrow and have a short half-life, whereas tissue-derived macrophages exist in the lungs from early embryonic development and survive for long periods through self-renewal [28–30].

The inhibitory effect of DSF on lymphocytic bronchiolitis observed in this study may contribute to the prevention of BO because lymphocytic bronchiolitis is regarded as its precursor lesion [31]. IL-6, a pro-inflammatory cytokine, is strongly implicated in acute rejection after lung transplantation [32, 33]. The suppression of IL-6 signaling reportedly inhibits the development of BO [34]. As the expression of *IL-6* in allografts was downregulated in the DSF group in our study, DSF may be able to inhibit BO. Furthermore, we also hypothesized that DSF suppresses inflammation in the bronchi because of the decrease in the TCC in the BALF. In the BO lesions of human lung tissue, phosphorylation-induced activation of nuclear factor (NF)-κB and STAT3 and an increase in the proportions of CD4^+^ T cells and macrophages have been reported [34]. As there is some evidence that DSF inhibits the NF-κB pathway [35, 36], it can be expected to prevent not only acute lung rejection but also BO and subsequent CLAD development.

There were a few limitations to this study. First, the effect of administering DSF via the oral route was not investigated. To stabilize DSF concentrations in the blood and prevent aspiration related to dosing and handling, we implanted osmotic pumps and administered the drug solutions subcutaneously. Second, the immune system varies from species to species. In this study, we employed the combination of F344 and Lewis rat strains, which is characterized by a minor mismatch in the MHC class I region. We acknowledge that this model does not fully represent the genetic diversity usually observed between human lung transplant donors and recipients. Furthermore, rodents, including the strains used in our study, are generally more likely to develop spontaneous tolerance compared to humans. However, we chose this model because it enables us to achieve relatively uniform levels of acute rejection within the same groups, without the complicating effects of intense post-transplant immunosuppression required in major mismatch models. Further studies are needed to confirm our findings in a large animal model before clinical trials. Third, this study focused on whether DSF can prevent acute lung rejection; therefore, the mechanism of drug action was not clarified. Although our group has previously revealed a part of its mechanism of action [12, 24], additional studies should be conducted to clarify its molecular mechanism in a rat lung transplantation model. The pharmacokinetics and safety profile of DSF are also well-known because the Food and Drug Administration approved it approximately 70 years ago [8].

In conclusion, DSF inhibited acute rejection after rat MiHC lung transplantation through an anti-immune response effect, especially involving macrophages. Targeting macrophages using DSF can be a new immunotherapeutic option to attenuate the rejection of allografts.

## Data Availability

The raw data supporting the conclusion of this article will be made available by the authors, without undue reservation.

## Footnotes

1
https://bioconductor.org/packages/release/bioc/html/DESeq2.html

2
https://www.rdocumentation.org/packages/stats/versions/3.6.2

3
https://www.rdocumentation.org/packages/RDAVIDWebService/versions/1.10.0

## Abbreviations

CD, cluster of differentiation; COVID-19, coronavirus disease 2019; HIV, human immunodeficiency virus; ISG, interferon-stimulated gene; PTX, pentraxin-related protein; RLE, relative log expression; BALF, bronchoalveolar lavage fluid; CCL, chemokine ligand; CLAD, chronic lung allograft dysfunction; DEG, differentially expressed gene; DSF, disulfiram; GAPDH, glyceraldehyde 3-phosphate dehydrogenase; HBC, hydroxypropyl-β-cyclodextrin; IL, interleukin; IFN-γ, interferon-gamma; MiHC, minor histocompatibility; NF, nuclear factor; TCC, total cell count; TNF-α, tumor necrosis factor α.

## References

[1] DeVito DA Hoffman LA Iacono AT Wells CL Grgurich W Zullo TG Pattern and Predictors of Early Rejection After Lung Transplantation. Am J Crit Care (2003) 12:497–507. 10.4037/ajcc2003.12.6.497 14619355

[2] Hachem RR Khalifah AP Chakinala MM Yusen RD Aloush AA Mohanakumar T The Significance of a Single Episode of Minimal Acute Rejection After Lung Transplantation. Transplantation (2005) 80:1406–13. 10.1097/01.tp.0000181161.60638.fa 16340783

[3] Todd JL Neely ML Kopetskie H Sever ML Kirchner J Frankel CW Risk Factors for Acute Rejection in the First Year After Lung Transplant: A Multicenter Study. Am J Respir Crit Care Med (2020) 202:576–85. 10.1164/rccm.201910-1915OC 32379979 PMC7427399

[4] Hirschburger M Zakrzewicz A Kummer W Padberg W Grau V. Nicotine Attenuates Macrophage Infiltration in Rat Lung Allografts. J Heart Lung Transpl (2009) 28:493–500. 10.1016/j.healun.2009.02.005 19416779

[5] Schmidt A Sucke J Fuchs-Moll G Freitag P Hirschburger M Kaufmann A Macrophages in Experimental Rat Lung Isografts and Allografts: Infiltration and Proliferation *In Situ* . J Leukoc Biol (2007) 81:186–94. 10.1189/jlb.0606377 17053164

[6] Chiu S Bharat A. Role of Monocytes and Macrophages in Regulating Immune Response Following Lung Transplantation. Curr Opin Organ Transpl (2016) 21:239–45. 10.1097/MOT.0000000000000313 PMC4858348 26977996

[7] Moshkelgosha S Duong A Wilson G Andrews T Berra G Renaud-Picard B Interferon-Stimulated and Metallothionein-Expressing Macrophages Are Associated With Acute and Chronic Allograft Dysfunction After Lung Transplantation. J Heart Lung Transpl (2022) 41:1556–69. 10.1016/j.healun.2022.05.005 35691795

[8] Suh JJ Pettinati HM Kampman KM O’Brien CP. The Status of Disulfiram: A Half of a Century Later. J Clin Psychopharmacol (2006) 26:290–302. 10.1097/01.jcp.0000222512.25649.08 16702894

[9] Hu JJ Liu X Xia S Zhang Z Zhang Y Zhao J FDA-Approved Disulfiram Inhibits Pyroptosis by Blocking Gasdermin D Pore Formation. Nat Immunol (2020) 21:736–45. 10.1038/s41590-020-0669-6 32367036 PMC7316630

[10] Custodio MM Sparks J Long TE. Disulfiram: A Repurposed Drug in Preclinical and Clinical Development for the Treatment of Infectious Diseases. Antiinfect Agents (2022) 20:e040122199856. 10.2174/2211352520666220104104747 35782673 PMC9245773

[11] Kona FR Buac D Burger AM. Disulfiram, and Disulfiram Derivatives as Novel Potential Anti-Cancer Drugs Targeting the Ubiquitin-Proteasome System in Both Preclinical and Clinical Studies. Curr Cancer Drug Targets (2011) 11:338–46. 10.2174/156800911794519798 21247383

[12] Terashima Y Toda E Itakura M Otsuji M Yoshinaga S Okumura K Targeting FROUNT With Disulfiram Suppresses Macrophage Accumulation and Its Tumor-Promoting Properties. Nat Commun (2020) 11:609. 10.1038/s41467-020-14338-5 32001710 PMC6992764

[13] Toda E Sawada A Takeuchi K Wakamatsu K Ishikawa A Kuwahara N Inhibition of the Chemokine Signal Regulator FROUNT by Disulfiram Ameliorates Crescentic Glomerulonephritis. Kidney Int (2022) 102:1276–90. 10.1016/j.kint.2022.07.031 36049642

[14] Ashburn TT Thor KB. Drug Repositioning: Identifying and Developing New Uses for Existing Drugs. Nat Rev Drug Discov (2004) 3:673–83. 10.1038/nrd1468 15286734

[15] ClinicalTrials. ClinicalTrials.gov (2023). Available from: https://clinicaltrials.gov/search?intr=Disulfiram (Accessed October 3, 2023).

[16] Tian D Shiiya H Sato M Nakajima J. Rat Lung Transplantation Model: Modifications of the Cuff Technique. Ann Transl Med (2020) 8:407. 10.21037/atm.2020.02.46 32355851 PMC7186686

[17] Stewart S Fishbein MC Snell GI Berry GJ Boehler A Burke MM Revision of the 1996 Working Formulation for the Standardization of Nomenclature in the Diagnosis of Lung Rejection. J Heart Lung Transpl (2007) 26:1229–42. 10.1016/j.healun.2007.10.017 18096473

[18] Bustin SA Benes V Garson JA Hellemans J Huggett J Kubista M The MIQE Guidelines: Minimum Information for Publication of Quantitative Real-Time PCR Experiments. Clin Chem (2009) 55:611–22. 10.1373/clinchem.2008.112797 19246619

[19] Jin Z Du X Xu Y Deng Y Liu M Zhao Y Structure of M(Pro) From SARS-CoV-2 and Discovery of Its Inhibitors. Nature (2020) 582:289–93. 10.1038/s41586-020-2223-y 32272481

[20] Rogers NJ Lechler RI. Allorecognition. Am J Transpl (2001) 1:97–102. 10.1034/j.1600-6143.2001.10201.x 12099369

[21] Sivaganesh S Harper SJ Conlon TM Callaghan CJ Saeb-Parsy K Negus MC Copresentation of Intact and Processed MHC Alloantigen by Recipient Dendritic Cells Enables Delivery of Linked Help to Alloreactive CD8 T Cells by Indirect-Pathway CD4 T Cells. J Immunol (2013) 190:5829–38. 10.4049/jimmunol.1300458 23630361 PMC3736307

[22] Mosser DM Edwards JP. Exploring the Full Spectrum of Macrophage Activation. Nat Rev Immunol (2008) 8:958–69. 10.1038/nri2448 19029990 PMC2724991

[23] Oyaizu T Okada Y Shoji W Matsumura Y Shimada K Sado T Reduction of Recipient Macrophages by Gadolinium Chloride Prevents Development of Obliterative Airway Disease in a Rat Model of Heterotopic Tracheal Transplantation. Transplantation (2003) 76:1214–20. 10.1097/01.TP.0000088672.48259.F1 14578756

[24] Toda E Terashima Y Sato T Hirose K Kanegasaki S Matsushima K. FROUNT Is a Common Regulator of CCR2 and CCR5 Signaling to Control Directional Migration. J Immunol (2009) 183:6387–94. 10.4049/jimmunol.0803469 19841162

[25] Maus UA Wellmann S Hampl C Kuziel WA Srivastava M Mack M CCR2-Positive Monocytes Recruited to Inflamed Lungs Downregulate Local CCL2 Chemokine Levels. Am J Physiol Lung Cel Mol Physiol (2005) 288:L350–8. 10.1152/ajplung.00061.2004 15516494

[26] Gelman AE Okazaki M Sugimoto S Li W Kornfeld CG Lai J CCR2 Regulates Monocyte Recruitment as Well as CD4 T1 Allorecognition After Lung Transplantation. Am J Transpl (2010) 10:1189–99. 10.1111/j.1600-6143.2010.03101.x PMC3746750 20420631

[27] Adrover JM Carrau L Daßler-Plenker J Bram Y Chandar V Houghton S Disulfiram Inhibits Neutrophil Extracellular Trap Formation and Protects Rodents From Acute Lung Injury and SARS-CoV-2 Infection. JCI Insight (2022) 7:e157342. 10.1172/jci.insight.157342 35133984 PMC8983145

[28] Landsman L Jung S. Lung Macrophages Serve as Obligatory Intermediate Between Blood Monocytes and Alveolar Macrophages. J Immunol (2007) 179:3488–94. 10.4049/jimmunol.179.6.3488 17785782

[29] Misharin AV Morales-Nebreda L Mutlu GM Budinger GR Perlman H. Flow Cytometric Analysis of Macrophages and Dendritic Cell Subsets in the Mouse Lung. Am J Respir Cel Mol Biol (2013) 49:503–10. 10.1165/rcmb.2013-0086MA PMC3824047 23672262

[30] Yona S Kim KW Wolf Y Mildner A Varol D Breker M Fate Mapping Reveals Origins and Dynamics of Monocytes and Tissue Macrophages Under Homeostasis. Immunity (2013) 38:79–91. 10.1016/j.immuni.2012.12.001 23273845 PMC3908543

[31] Glanville AR Aboyoun CL Havryk A Plit M Rainer S Malouf MA. Severity of Lymphocytic Bronchiolitis Predicts Long-Term Outcome After Lung Transplantation. Am J Respir Crit Care Med (2008) 177:1033–40. 10.1164/rccm.200706-951OC 18263803

[32] Iacono A Dauber J Keenan R Spichty K Cai J Grgurich W Interleukin 6 and Interferon-Gamma Gene Expression in Lung Transplant Recipients With Refractory Acute Cellular Rejection: Implications for Monitoring and Inhibition by Treatment With Aerosolized Cyclosporine. Transplantation (1997) 64:263–9. 10.1097/00007890-199707270-00015 9256185

[33] Yoshida Y Iwaki Y Pham S Dauber JH Yousem SA Zeevi A Benefits of Posttransplantation Monitoring of Interleukin 6 in Lung Transplantation. Ann Thorac Surg (1993) 55:89–93. 10.1016/0003-4975(93)90479-2 8417717

[34] Lee J Nakagiri T Kamimura D Harada M Oto T Susaki Y IL-6 Amplifier Activation in Epithelial Regions of Bronchi After Allogeneic Lung Transplantation. Int Immunol (2013) 25:319–32. 10.1093/intimm/dxs158 23396843

[35] Celik O Ersahin A Acet M Çelik N Baykuş Y Deniz R Disulfiram, as a Candidate NF-Κb and Proteasome Inhibitor, Prevents Endometriotic Implant Growing in a Rat Model of Endometriosis. Eur Rev Med Pharmacol Sci (2016) 20:4380–9. 27831632

[36] Zha J Chen F Dong H Shi P Yao Y Zhang Y Disulfiram Targeting Lymphoid Malignant Cell Lines Via ROS-JNK Activation as Well as Nrf2 and NF-kB Pathway Inhibition. J Transl Med (2014) 12:163. 10.1186/1479-5876-12-163 24915933 PMC4075939

