# Supplementary material for: Disulfiram, an Anti-alcoholic Drug, Targets Macrophages and Attenuates Acute Rejection in Rat Lung Allografts
Source: Transpl Int. 2024 Apr 8;37:12556. doi: 10.3389/ti.2024.12556 (PMC11033352; doi:10.3389/ti.2024.12556)
Supplement: Supplementary file 2 [file DataSheet7.docx]

**Disulfiram,** **an Anti-alcoholic Drug, Targets Macrophages and Attenuates Acute Rejection in Rat Lung Allografts**

Nobuyuki Yoshiyasu, MD, PhD^1^; Rei Matsuki, MD, PhD^2^; Masaaki Sato, MD, PhD^3^*; Hirokazu Urushiyama, MD, PhD^4^; Etsuko Toda, PhD^5,6^; Yasuhiro Terasaki, MD, PhD^5,7^; Masaki Suzuki, MD, PhD^8^; Aya Shinozaki-Ushiku, MD, PhD^8^; Yuya Terashima, PhD^6^; Jun Nakajima, MD, PhD^3^

^1^Department of Thoracic Surgery, Graduate School of Medicine, The University of Tokyo, Tokyo, Japan

^2^Department of Respiratory Medicine, Graduate School of Medicine, The University of Tokyo, Tokyo, Japan

^3^Department of Thoracic Surgery, The University of Tokyo Hospital, Tokyo, Japan

^4^Department of Respiratory Medicine, The University of Tokyo Hospital, Tokyo, Japan

^5^Department of Analytic Human Pathology, Nippon Medical School, Tokyo, Japan

^6^Division of Molecular Regulation of Inflammatory and Immune Diseases, Research Institute for Biomedical Sciences (RIBS), Tokyo University of Science, Chiba, Japan

^7^Division of Pathology, Nippon Medical School Hospital, Tokyo, Japan

^8^Department of Pathology, The University of Tokyo Hospital, Tokyo, Japan

**TABLE OF CONTENTS**

Page

Supplementary Figure 1. Gene expression analyses using RNA sequencing3

Supplementary Figure 2. Absolute numbers of macrophages, lymphocytes, and neutrophils in the bronchoalveolar lavage fluid (cells/mL)4


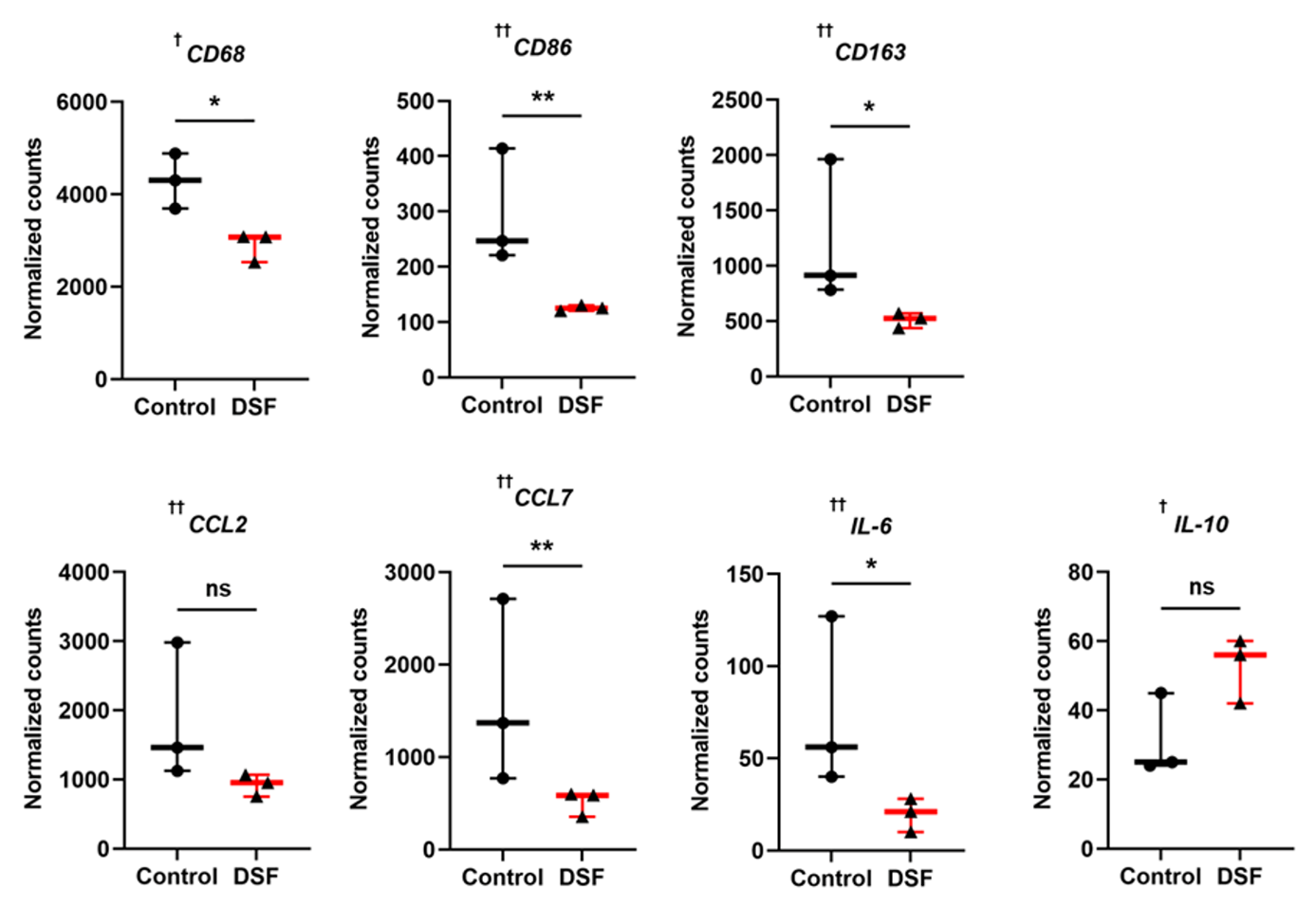


**Supplementary Figure 1. Gene expression analyses using RNA sequencing.**

RNA expression levels in the two groups (n = 3/group) are demonstrated. The data reveal the normalized expression of each gene using the DESeq2 package. The box-and-whiskers dot plots represent the medians and interquartile ranges with the minimum and maximum values. The Benjamini–Hochberg method was used for analysis. ns, not significant. * *Q* < 0.05; ** *Q* < 0.01. ^†^|log_2_ fold change| > 0.6. ^††^|log_2_ fold change| > 1.


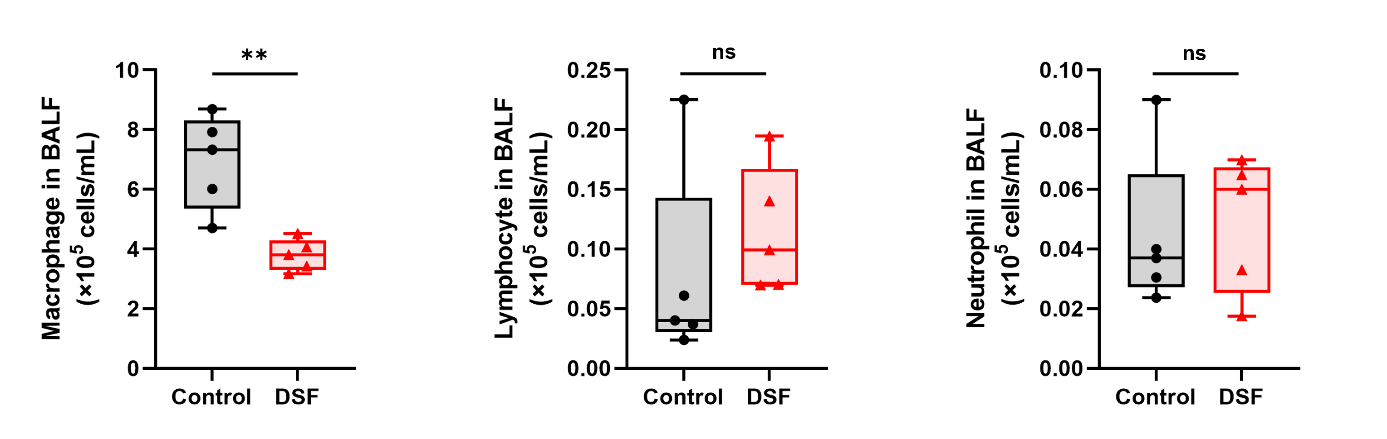


**Supplementary Figure 2. Absolute numbers of macrophages, lymphocytes, and neutrophils in the bronchoalveolar lavage fluid (cells/mL).** The box-and-whiskers dot plots represent the medians and interquartile ranges with the minimum and maximum values. ns, not significant. ** *P* < 0.01. DSF, disulfiram.
